# Hereditary multiple osteochondromas in a child: a case report and discussion of postoperative complication management

**DOI:** 10.3389/fsurg.2025.1689110

**Published:** 2025-11-07

**Authors:** Haiting Jia, Yuting Wang, Tao Liu

**Affiliations:** Department of Orthopaedic Trauma Surgery, Children’s Hospital Affiliated to Shandong University (Jinan Children’s Hospital), Jinan, Shandong, China

**Keywords:** hereditary multiple osteochondromas, *EXT1* gene, surgery, femoral artery rupture, child

## Abstract

**Background:**

The pathogenesis of hereditary multiple exostoses is mainly related to genetic variants and often requires surgical resection when it causes clinical symptoms. This case report describes a variant in the *EXT1* gene and the management of postoperative femoral artery rupture.

**Case presentation:**

We present the case of an 11-year-old boy who developed hereditary multiple exostoses. The patient presented with multiple bone swellings throughout his body and difficulty squatting due to a swelling in his right thigh. Genetic testing showed that the child had a heterozygous variant in the *EXT1* gene c.1722+1G>A (p.?). We performed a resection of the osteochondroma of the right femur; however, after the surgery, there was persistent bleeding from the wound. Surgical exploration revealed a rupture of the right femoral artery, which we repaired.

**Conclusions:**

The diagnosis of hereditary multiple exostoses relies on a clinical examination and genetic testing. Surgical resection is indicated for symptomatic cases with functional impairments. To prevent vascular injuries such as femoral artery rupture, meticulous surgical technique is essential, including thorough smoothing of the resected bone surface and a careful intraoperative assessment of the adjacent neurovascular structures. In cases of postoperative bleeding or suspected pseudoaneurysm, prompt imaging and surgical exploration are critical for timely vascular repair.

## Introduction

Hereditary multiple osteochondromas is an autosomal dominant disease involving cartilaginous bone and is characterized by multiple osteochondromas (1). The disease was first genetically studied in 1964 by Solomon (2). Osteochondroma formation is mainly caused by an abnormal proliferation of chondrocytes at the epiphyseal plate, where a cartilaginous cap covers the surface and acts as an epiphyseal growth plate (3). The main clinical manifestation of hereditary multiple osteochondromas is a bony mass in the epiphysis that can cause pain, deformity, and limited joint movement. Heterozygous loss-of-function variants in the *EXT1* and *EXT2* genes are the primary cause of hereditary multiple osteochondromas and are identified in the majority of reported cases (1). This case report describes a variant in the *EXT1* gene and the management of a femoral artery rupture following an osteochondroma resection.

## Case presentation

We present the case of an 11-year-old boy with multiple bone swellings throughout his body for 7 years. The swellings were first noted at the age of 4 years, but the child experienced no discomfort until the age of 9, when a bone mass on the back of his right thigh increased in size and caused difficulty in squatting. The child's father has multiple osteochondromas, while the child's sister and mother are in good health.

Upon examination, the patient had multiple palpable swellings on his extremities, which were hard and nontender. These swellings were located at the metaphyses of the bilateral ulnae and radii, the bilateral carpal bones, the distal left humerus, the metaphyses of the bilateral femurs, and the metaphyses of the bilateral tibias and fibulas. Among these, a larger swelling on the posterior aspect of the right thigh, measuring approximately 5 cm × 4 cm × 3 cm, was noted, which limited the patient’s squatting. The remaining affected areas showed no significant deformities, and the masses did not impair joint mobility or cause notable neurological symptoms.

**Figure 1:**
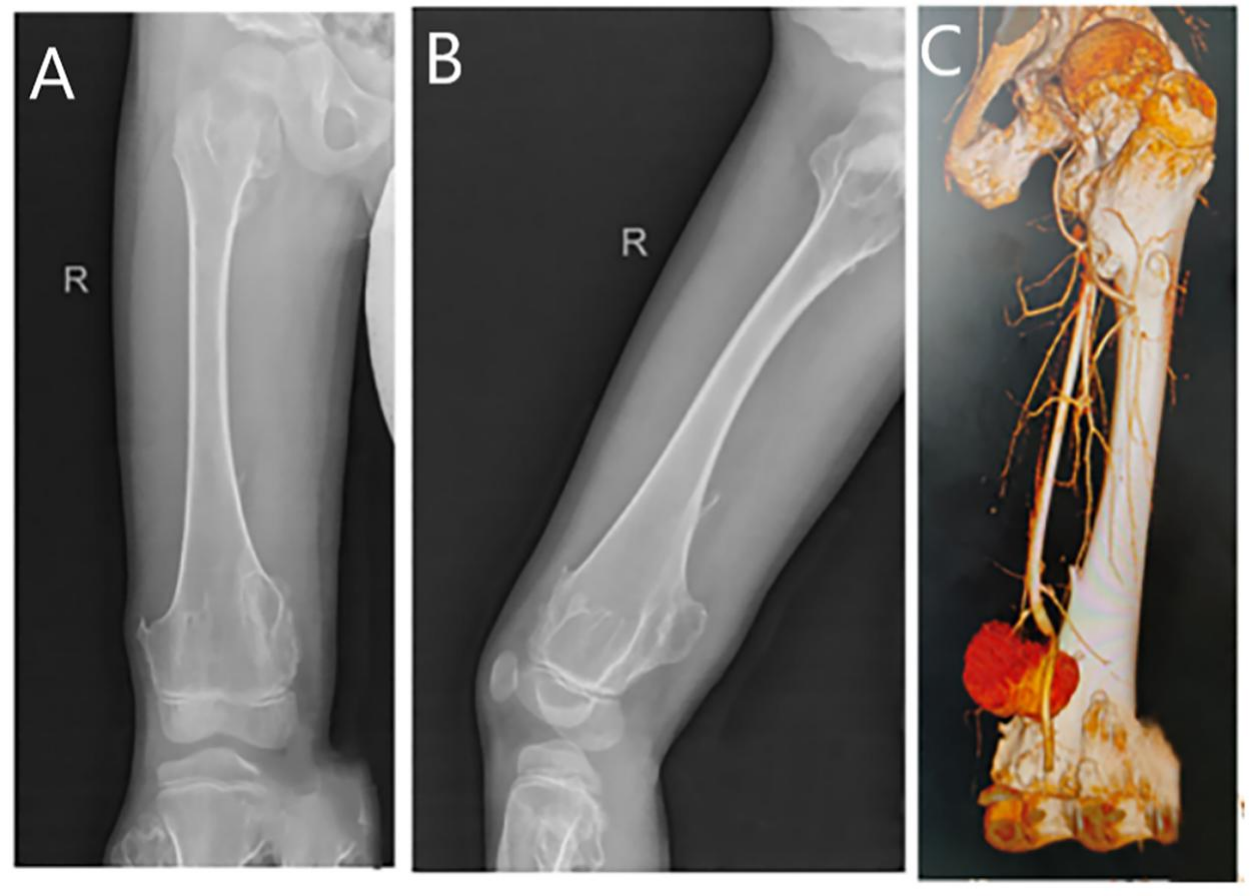
**(A)** A plain radiograph in the anterior–posterior view reveals an osteochondroma of the right distal femur. **(B)** A lateral view of the same plain radiograph reveals an osteochondroma of the right distal femur. **(C)** Computed tomography angiography shows a distal rupture of the right femoral artery with pseudoaneurysm formation.

A— radiograph was performed, and genetic testing was sent for analysis. The radiograph showed an osteochondroma of the right distal femur (Figures 1A,B). Whole-exome sequencing analysis revealed a pathogenic heterozygous variant in the *EXT1* gene (NM_000127.3:c.1722+1G>A) in intron 8. Sanger sequencing confirmed paternal inheritance of this variant, while the patient’s sister and mother carried the wild-type allele.

We excised the mass from the back of the patient’s right thigh, and postoperative pathology confirmed that it was an osteochondroma. On the 10th postoperative day, the patient experienced a partial dehiscence of the surgical incision, accompanied by bleeding. The initial discharge was dark red in color, which subsequently transitioned to bright red and ultimately evolved into hematoma formation. Laboratory tests revealed a red blood cell count of 3.46 × 10^12^/L, a hemoglobin level of 102 g/L, and a hematocrit of 30.4%, all of which were below the normal reference ranges. An ultrasound examination revealed a cystic mass measuring approximately 14.9 cm × 6.8 cm × 4.3 cm located within the subcutaneous muscular layer. Internal blood flow signals were detected within the mass via Doppler imaging. The deep aspect of the lesion was adjacent to the right femoral artery, with an echogenic finding consistent with a fistulous tract approximately 0.18 cm in width. These findings were suggestive of a pseudoaneurysm arising from the right femoral artery, accompanied by surrounding intramuscular hematoma formation. Further computed tomography angiography revealed a sharp and rough local cortical bone at the distal end of the right femur. An irregular mass shadow was seen on the dorsal side of the lower segment of the right thigh, with a relatively thick cyst wall. Enhanced scanning revealed that the contrast agent was filling the inside of the cyst. This indicated a rupture at the distal end of the right artery and the formation of a pseudoaneurysm (Figure 1C).

We performed a surgical repair of the ruptured femoral artery. The original incision at the posterior aspect of the distal right thigh was reused, and the distal femoral artery was gradually exposed. Intraoperatively, a 1.5 cm × 0.3 cm defect was noted on the femoral arterial wall, and the adjacent femoral cortical bone had sharp, rough edges, which were trimmed. A biological patch was then cut to size, placed over the arterial defect, and secured with continuous sutures. Post-repair, the femoral artery defect was successfully repaired, with good pulsation preserved.

## Discussion

Hereditary multiple osteochondromas represent an autosomal dominant disorder with an incidence of approximately 1/50,000 (4). Hereditary multiple osteochondromas are commonly found in the metaphysis of long bones, but can also involve the ribs, scapulae, and vertebrae (5). Hereditary multiple osteochondromas are caused by heterozygous loss-of-function variants in the *EXT1* and *EXT2* genes, with approximately 65% of cases involving *EXT*1 and 21% involving *EXT2* (6).

Hereditary multiple osteochondromas present as multiple exophytic bony warts and are generally not managed in asymptomatic individuals, whereas surgery is recommended for those with symptoms such as deformity, limited mobility, and pain (4). The removal of osteochondromas must include the cartilage cap and the overlying perichondrium to prevent recurrence. Forearm deformities are managed by removing exostoses, performing corrective osteotomies, and lengthening the ulna. Leg length differences over 2.5 cm are often addressed by stopping growth in the longer leg or lengthening the shorter one. Angular misalignment in the lower limbs can be corrected with growth plate surgery (hemiepiphysiodesis) or osteotomies in the knee or ankle. Early intervention for ankle deformities can prevent future functional decline. Surgical removal is the treatment for sarcomatous degeneration (7). This case presented with squatting difficulties in the right lower extremity, without other significant symptoms, and met the surgical indications for excision. The remaining affected areas showed no significant deformities, and the masses did not impair his joint mobility or cause notable neurological symptoms. Therefore, surgical intervention was not performed on these masses at this time. However, this case presented with wound bleeding and hematoma formation after surgery, which was confirmed as a femoral artery pseudoaneurysm by ultrasound and computed tomography angiography.

The incidence of femoral artery pseudoaneurysms ranges from approximately 0.06% to 7.7%, with the majority of these secondary to medical and traumatic injuries, of which medical injury is the most common etiology (8). After rupture of the femoral artery, blood enters the surrounding tissues to form a pulsatile hematoma, which forms a pseudoaneurysm that is compressed by the surrounding tissues. Delayed diagnosis and treatment of a femoral artery pseudoaneurysm can lead to peripheral nerve compression, distal limb ischemia, thromboembolism, and osteofascial compartment syndrome (8, 9).

Current treatments for a femoral artery pseudoaneurysm include conservative, surgical, and interventional approaches. Conservative treatment involves placing pressure on the wound to stop the bleeding and waiting for it to heal naturally. Surgery is the traditional treatment and includes the resection of the pseudoaneurysm and repair of the arterial rupture. In recent years, interventional therapies, including ultrasound-guided thrombin injection therapy and endovascular therapy, have been used more frequently (9–11). In this case, the femoral artery pseudoaneurysm occurred at the site of the surgical resection and was considered to be caused by the sharp and rough bone that injured the femoral artery after the resection of the osteochondroma. Given the need to simultaneously repair the rough and sharp bony surfaces, interventional treatment was not suitable; therefore, surgical treatment was chosen to maximize the repair of the surfaces to help prevent re-injury.

While osteochondromas are predominantly benign and asymptomatic, their potential to cause vascular complications, such as pseudoaneurysms, warrants a proactive and preventive approach to management. To mitigate these rare but serious risks, several practical strategies should be emphasized. First, the early identification of high-risk lesions is crucial. Osteochondromas located in proximity to major neurovascular bundles, particularly those with a pedunculated or sharp morphology at the femoro-popliteal junction, should be considered high-risk, and a preventive resection is recommended (12). Second, vigilant monitoring through multimodal imaging is recommended. Ultrasound, radiographs, and computed tomography angiography are necessary for the preoperative assessment (12, 13). Finally, the case reported in this article underscores the importance of meticulous surgical technique, including thorough smoothing of the resected bone surface and a careful intraoperative assessment of the adjacent neurovascular structures.

## Conclusion

We reported a case of hereditary multiple osteochondromas with a pathogenic *EXT1* gene variant. Surgical intervention is recommended for symptomatic lesions that cause functional limitations. This case highlights the importance of intraoperative precautions to prevent vascular injury, such as meticulous bone surface smoothing and the protection of adjacent vessels. In the event of a postoperative hemorrhage or pseudoaneurysm formation, an early diagnosis via ultrasound or computed tomography angiography and prompt surgical repair are essential to prevent serious complications.

## Data Availability

The original contributions presented in the study are included in the article/Supplementary Material, further inquiries can be directed to the corresponding author.
